# Cytomegalovirus Colitis Mimicking Ulcerative Colitis Flare in an Immunocompromised Patient: A Challenging Diagnosis

**DOI:** 10.7759/cureus.71099

**Published:** 2024-10-08

**Authors:** Abdul Moiz Khan, Amna Ehtisham, Humais Habib Choudhary

**Affiliations:** 1 Internal Medicine, Sahiwal Medical College, Sahiwal, PAK

**Keywords:** antiviral drugs, bloody diarrhea, cytomegalovirus (cmv), immunocompromise, ulcerative colitis

## Abstract

This case report details a 40-year-old male patient with a background of ulcerative colitis (UC), who presented with persistent bloody diarrhea refractory to standard treatment. The clinical picture was initially suggestive of a UC flare-up, prompting the continued use of immunosuppressive therapy. However, histopathological evaluation ultimately revealed cytomegalovirus (CMV) colitis, a condition that can mimic UC exacerbation but requires a distinct treatment approach. The patient’s immunosuppressive therapy, essential for UC management, likely contributed to the reactivation of latent CMV infection, underscoring the complexity of managing such cases.

This case underscores the critical importance of a multidisciplinary approach in differentiating between UC exacerbation and opportunistic infections such as CMV colitis, especially in immunocompromised patients.

## Introduction

Ulcerative colitis (UC) is a chronic inflammatory bowel disease characterized by recurrent episodes of mucosal inflammation confined to the colon and rectum. Its hallmark is ulcers (sores) in the innermost lining of the colon. Although the exact cause of UC is unknown, it is believed to be caused by an abnormal immune response, possibly triggered by environmental factors, genetic predisposition, or an imbalance in the gut microbiome. UC is a relapsing-remitting condition, meaning patients can experience periods of flare-ups and remission [1]. Despite significant advancements in the treatment of UC, managing this condition remains challenging, often necessitating the use of immunosuppressive therapies to control symptoms and prevent disease progression [2]. However, these immunosuppressive agents also predispose patients to opportunistic infections, complicating the clinical picture. As patients with UC are more likely to experience opportunistic infections when receiving immunosuppressive medications, it is critical to diagnose and treat infectious consequences in a clinical environment [3]. In the context of UC, cytomegalovirus (CMV) colitis can be a serious complication, especially during a flare-up or when patients are being treated with immunosuppressive medications such as corticosteroids or biologics [3].  In adult patients with moderate-to-severe UC, CMV colitis is observed in 25-30%, especially those exhibiting steroid-refractory disease [4]. In one study, colonic mucosal CMV disease detected by histopathology was associated with intravenous steroid resistance in 5-36% compared to 0-10% of steroid-responsive patients. CMV colitis has rarely been reported in association with UC without steroids or other immunomodulators [5]. CMV can target mainly immunocompromised individuals and can cause a variety of symptoms which can include esophagitis and colitis. For effective and prompt intervention in individuals with UC, it is crucial to differentiate between a superimposed CMV infection and an aggravation of the underlying inflammatory bowel disease [6].

The simultaneous development of UC and CMV colitis presents treating physicians with a diagnostic dilemma. Therefore, an integrated approach is required for an accurate diagnosis and successful management of this condition [7]. Here, we describe a middle-aged man with a history of UC who first presented with symptoms indicative of an exacerbation, which turned out to be acute CMV colitis on histopathologic examination. This emphasizes the significance of taking opportunistic infections into account when making a differential diagnosis of exacerbations of inflammatory bowel disease.

## Case presentation

A 40-year-old male Asian patient presented to the emergency department of a tertiary care hospital with complaints of mild fever, multiple episodes of bloody diarrhea, and lethargy for the past two days. The patient did not have any past medical history of human immunodeficiency virus, tuberculosis, or any other medical disease. The patient previously had a controlled case of UC and did not require any inpatient admission during his follow-up period.

On admission, the patient had eight episodes of persistent bloody diarrhea and acute-onset abdominal pain lasting for two days and associated with frequent episodes of vomiting refractory to treatment. Upon physical examination, the patient appeared pale and underweight. Moreover, jaundice was not appreciable. The complete blood count (CBC) showed microcytic anemia (Table 1).

**Table 1:** Complete blood count of the patient done at the time of admission.

Test	Result	Reference range
White cell count	6,510	4,000–10,000/µL
Red blood cell count	4.06	4.5–5.5 million/µL
Hemoglobin	10.6	13–17 g/dL
Mean corpuscular volume	77.2	80–98 fL
Hematocrit	31.3	40–50%
Platelet count	311,000	150,000–400,000/µL
Differential leukocyte count		
Neutrophils	83.3	45–70%
Lymphocytes	11.2	20–40%
Eosinophils	0.5	1–6%
Basophils	0.2	0.5–1%
C-reactive protein	40	<5

Urinalysis and liver function tests were normal (Table 2). The stool sample for microscopy, sensitivity, and culture did not reveal any infective etiology.

**Table 2:** Liver function tests and urinalysis.

Conventional system			International system	
Test name	Result	Reference range	Result	Reference range
Bilirubin total	0.590	0.3–1.2 mg/dL	10.1	5.1–20.5 µmol/L
Alanine aminotransferase	17.1	4–42 U/L	17.1	4–42 U/L
Creatinine	1.05	0.6–1.3 mg/dL	92.8	53–114.9 µmol/L
Sodium	126.7	136–146 mEq/L	126.7	136–146 mmol/L
Alkaline phosphate	67.8	40–130 U/L	67.8	40–130 U/L
Urea	20.1	13–43 mg/dL	3.3	2.2–7.1 mmol/L

The patient was referred by the medical team for colonoscopy given his history of UC for the past five years that was controlled on infliximab and azathioprine combination therapy. His Truelove and Witts score on presentation classified him as having severe UC.

**Figure 1:**
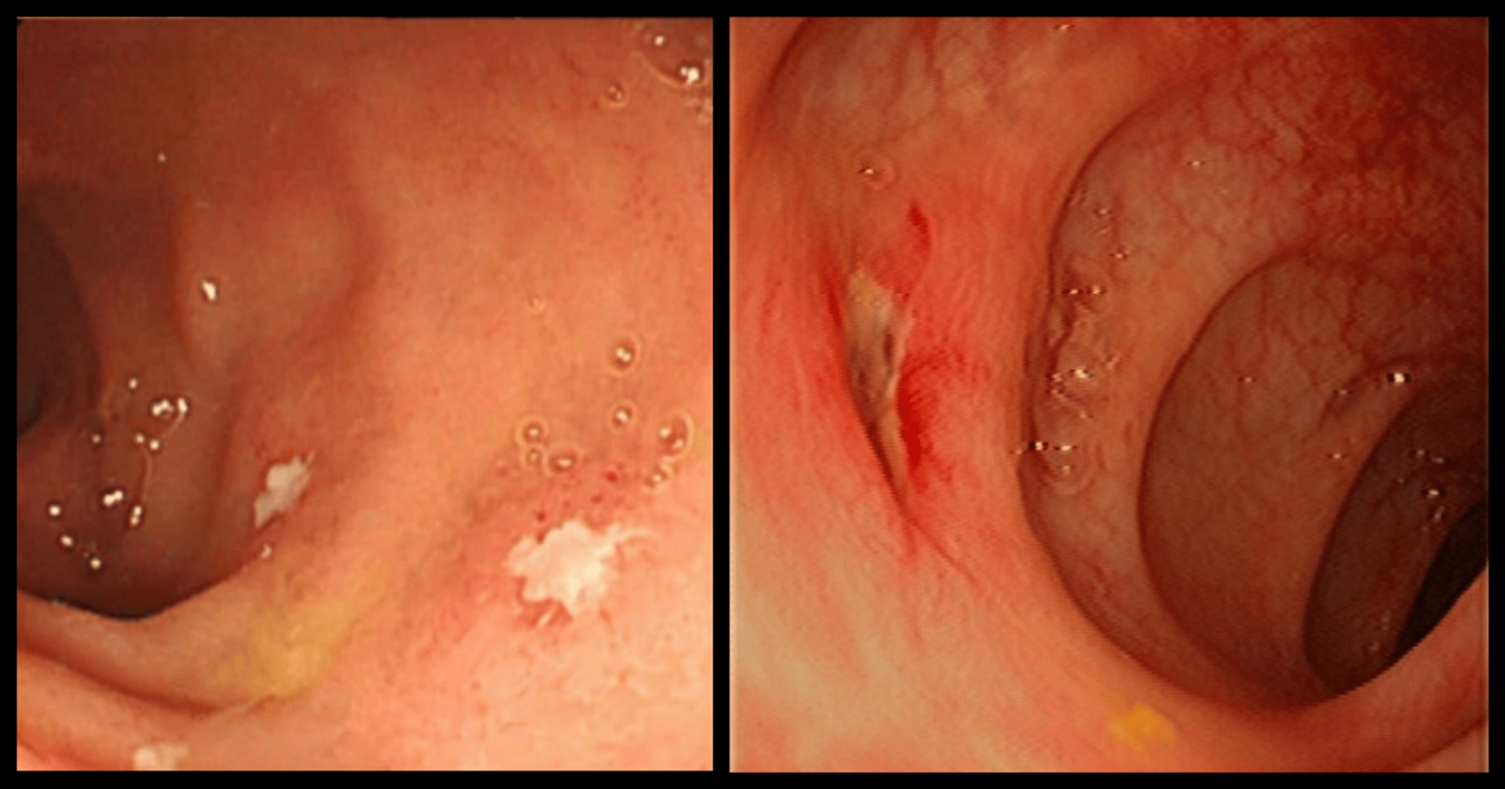
Colonoscopy showing active colitis with multiple deep ulcerations in the colon.

On colonoscopy, edematous mucosa, multiple deep ulcerations, and pseudo-polypoid formations were found in the rectum, sigmoid, and throughout the colon along with enlarged lymph nodes (Figure 1). No strictures were seen. However, small longitudinal ulcerations on the rectal margin were noted. Biopsy of the lymph nodes and further sections from the length of the intestine were sent for histopathologic examination.

**Figure 2:**
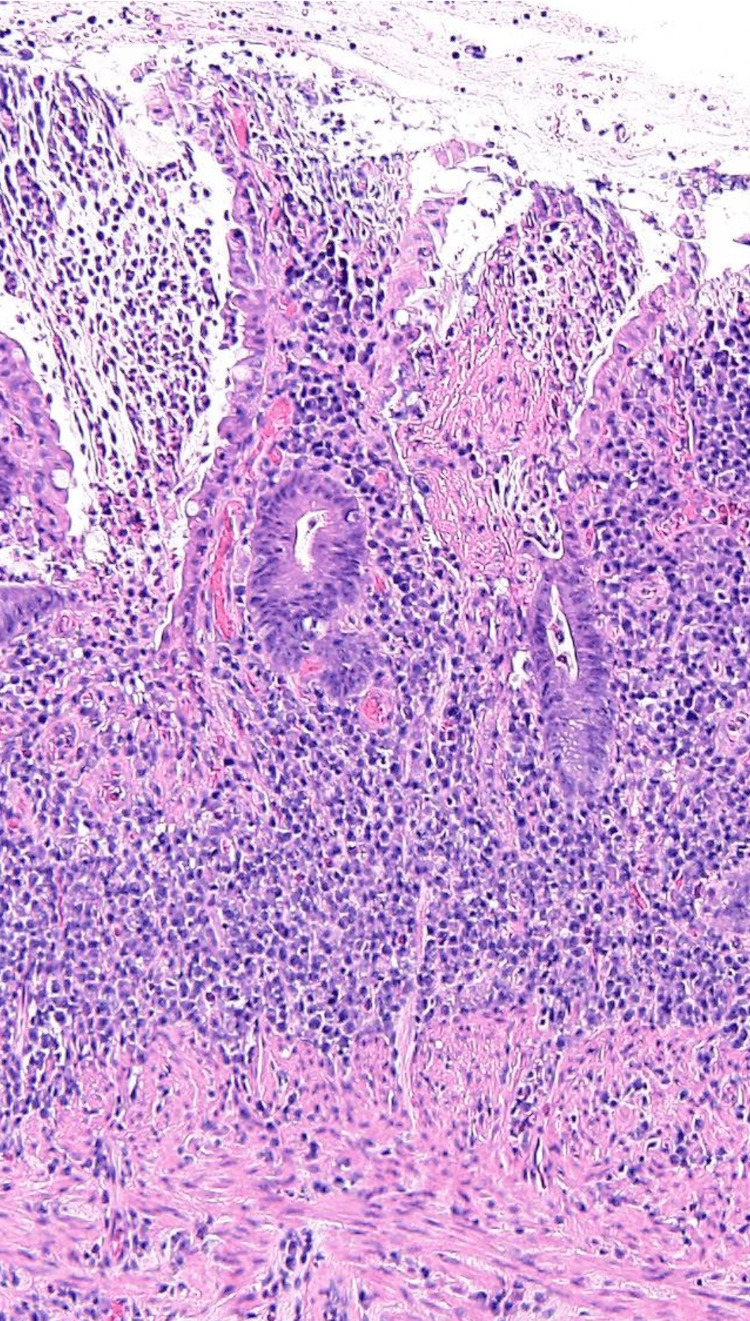
Microscopic image showing edematous intestinal mucosa, lack of normal folds, and the loss of tissue architecture.

The major findings on biopsy were the edematous intestinal mucosa, lacking normal folds, with multiple minute erosions, and the loss of tissue architecture (Figure 2).

The patient was diagnosed as having a flare episode of UC and was referred to the gastroenterology team for further management. The patient was started on intravenous (IV) hydrocortisone (100 mg four times a day) for five days while admitting the patient.

On day six, the symptoms had mildly improved as the frequency of diarrhea was reduced to three per day, and the associated symptoms were also relieved. He was tapered off of corticosteroids and was shifted to prednisolone 40 mg once a day.

On day eight, his symptoms started worsening as he experienced an increased frequency of bloody diarrhea. He was placed back on the previous IV hydrocortisone dose, but the symptoms persisted despite the treatment and posed ambiguity about our diagnosis of a flare episode of UC.

**Figure 3:**
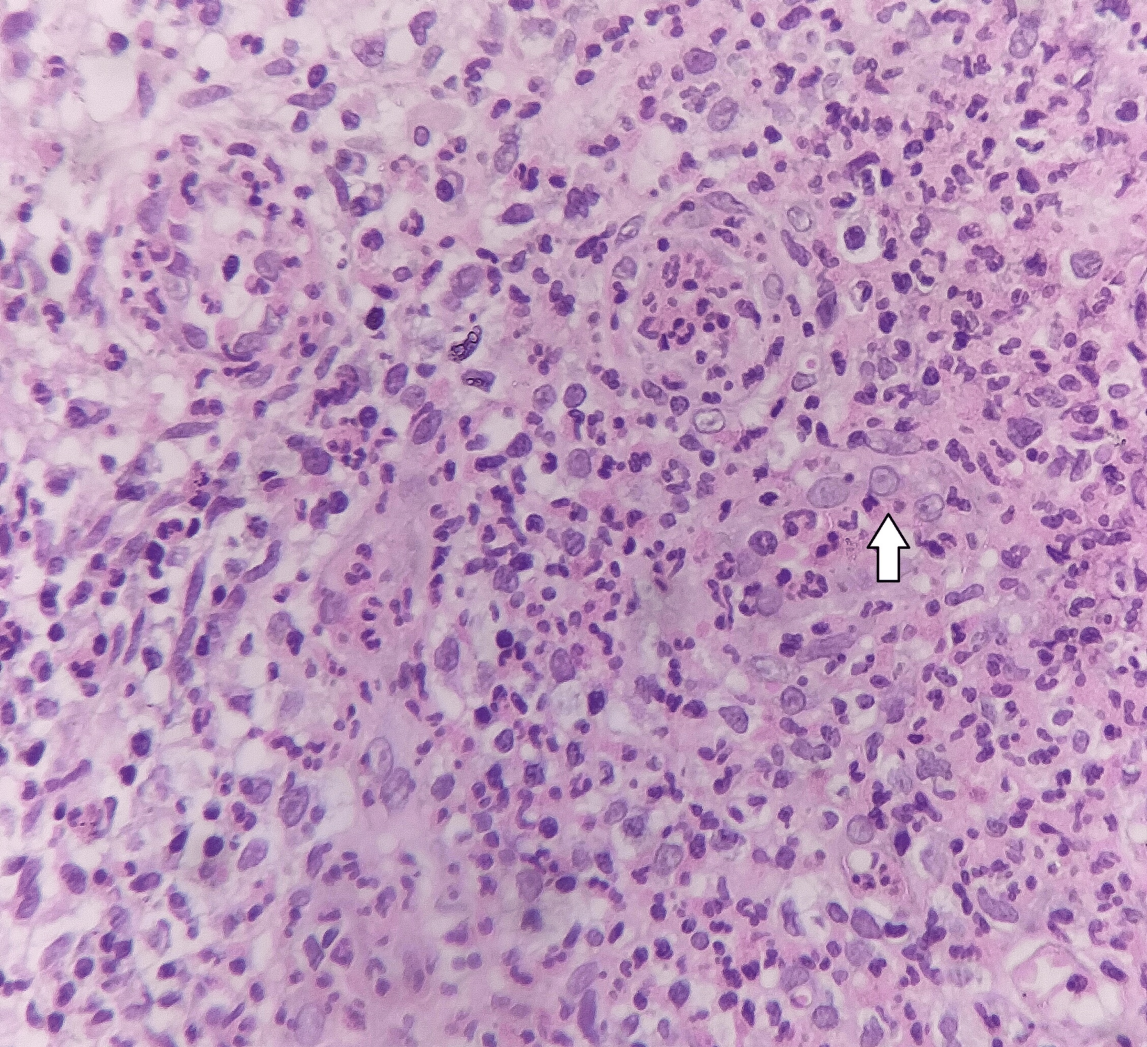
Hematoxylin and eosin staining showing cytomegalovirus intranuclear inclusion bodies.

On day 10, his histologic examination report revealed cells larger than normal with eccentrically placed bright pink intranuclear inclusions surrounded by a clear halo and the cytoplasm giving an “Owl’s-Eye” appearance (Figure 3). Apart from this, reactive lymphocytes were also found embedded in the lymph nodal tissue. These findings strongly pointed in the direction of a possible diagnosis of CMV colitis superimposed on UC. His viral polymerase chain reaction (PCR) for CMV revealed supportive evidence of CMV colitis superimposed on UC.

He was immediately taken off hydrocortisone and was started on oral valganciclovir 900 mg orally twice a day for three weeks. On day 14, the patient started improving and had no further episodes of bloody diarrhea. Valganciclovir dosage was continued for three weeks. He was discharged home with a routine dosage of 900 mg once daily. The patient was advised of monthly follow-up visits, and on routine visits, he showed no symptoms of CMV colitis and showed good signs of recovery.

## Discussion

CMV infection is only symptomatic when the immune system is compromised. The common manifestations of the disease include retinitis, encephalitis, and esophagitis. CMV colitis is an even rarer presentation. However, more and more cases of CMV colitis are being reported, and a substantial number of them are associated with UC [8,9].

The standard treatment regimen for UC includes corticosteroids, immunomodulators, and immune inhibitors, all of which are known to be immunosuppressive. The use of these drugs possibly increases the risk of developing a full-blown CMV infection in these patients [6].

The patient discussed in this report initially presented with bloody diarrhea and lethargy. After proper evaluation, he was diagnosed with an exacerbation of UC. He was started on hydrocortisone but had an underlying CMV colitis infection. Being on a stable corticosteroid regimen ameliorated the symptoms of UC but also immunocompromised the patient, which later led to the possible reactivation of a latent CMV infection.

This case highlights the ambiguity that arises when formulating a differential diagnosis for exacerbated symptoms in a known case of inflammatory bowel disease, specifically UC, which can hinder the provision of adequate and timely treatment.

The patient, who initially seemed to be experiencing a flare-up of UC, was found upon further testing to have a CMV infection superimposed on UC. The two conditions were confused due to their similar presentation patterns [10]. The signs and symptoms of UC include abdominal pain, diarrhea, and mucosal ulcerations. All of these symptoms are also reported in CMV colitis. It is important to differentiate between the two, as they require different courses of treatment. With vigilant observation, proper diagnostic tools, and timely intervention, the outcome is favorable.

## Conclusions

This case report highlights the complexities of diagnosing CMV infection in a patient with UC on treatment with immunosuppressants such as steroids. Due to the similar clinical presentations, it can be difficult to differentiate between an exacerbation of UC and a superimposed CMV infection. Laboratory investigations, such as biopsy and colonoscopy, are crucial for diagnosing CMV colitis and distinguishing it from a flare-up of UC as the CMV PCR can be negative for even invasive disease at times. This case serves as a useful reminder for physicians to remain vigilant for opportunistic infections in patients with inflammatory bowel disease, as this vigilance can prompt timely actions that enhance clinical outcomes.
